# Head and Neck Cancer Metastasis and the Effect of the Local Soluble Factors, from the Microenvironment, on Signalling Pathways: Is It All about the Akt?

**DOI:** 10.3390/cancers12082093

**Published:** 2020-07-28

**Authors:** Hanan Ahmed, Arpa Ghoshal, Sarah Jones, Ian Ellis, Mohammad Islam

**Affiliations:** School of Dentistry, University of Dundee, Dundee, Scotland DD1 4HR, UK; hanan.y.f.ahmed@gmail.com (H.A.); a.ghoshal@dundee.ac.uk (A.G.); s.j.jones@dundee.ac.uk (S.J.); i.r.ellis@dundee.ac.uk (I.E.)

**Keywords:** head and neck cancer, tumour microenvironment, cancer-associated fibroblasts, metastasis, cell migration, Akt

## Abstract

The signalling pathways involved in metastasis of oral adenoid cancer cells (TYS) in response to cancer-associated fibroblasts (COM D24) and normal oral mucosal fibroblasts (MM1) was examined. Metastatic cell behaviour was observed by cell-scatter, 3-D-collagen gel migration, and 3-D-spheroid invasion assays. Akt (v-Akt murine thymoma viral oncogene), MAPK(Mitogen activated protein kinase), EGFR (Epidermal growth factor receptor), TGFβRI (Transforming growth factor beta receptor 1), and CXCR4 (C-X-C chemokine receptor 4) inhibitors were used to identify the signalling pathways involved. Signalling pathway protein expression and activation were assessed by SDS-PAGE and Western blotting. COM-CM (conditioned medium from COM D24 cells) and MM1-CM (conditioned medium from MM1 cells) stimulated cancer cell scattering, which was blocked only by the Akt inhibitor. COM-CM-induced scattered cancer cells showed higher levels of Akt phosphorylation than the negative control and MM1-CM. Migration and invasion of TYS cells into collagen gels from the spheroids was stimulated by CM from both fibroblast cell lines, compared to the negative control. COM cells stimulated TYS invasion into the collagen more than MM1 and the control. Akt and EGFR inhibitors effectively blocked CM and COM cell-induced invasion. Akt-silenced cancer cells were not stimulated to migrate and invade by fibroblast-CM and did not survive the addition of an EGFR inhibitor. This suggests that CAFs stimulate head and neck cancer cell migration and invasion in an Akt- dependent manner. Akt may represent a potential target for inhibitor design to treat metastatic head and neck cancer.

## 1. Introduction

Head and neck cancers are cancers of the lip and oral cavity, salivary gland, larynx, and pharynx. It is the seventh most common cancer worldwide, with more than 887,000 cases and 450,000 deaths every year (accumulation of different head and neck cancer sites) [1]. Despite advances in diagnosis and treatment for head and neck cancer, the poor survival of patients has not fundamentally changed, mainly because of its high recurrence rate and strong inclination to metastasise [2]. Emerging evidence has found that the context in which malignant cells reside, now known as the tumour microenvironment (TME), has been implicated in orchestrating cancer survival and progression, through the continuous cross-interaction between cancer cells and the stroma [3,4,5]. Thus, the concept that a tumour is only a cancer-cell-centred disease has developed and it is now understood that there are complex multicellular interactions between cancer and stromal cells.

In normal tissue, fibroblasts are usually found in a quiescent state and become ‘transiently’ activated in certain physiological conditions, such as wound healing. However, in the tumour microenvironment, fibroblasts, which are the predominantly stromal cells, are perpetually activated without reverting to their normal state or undergoing apoptosis, and they have been termed cancer-associated fibroblasts (CAFs) [6,7]. Several studies have recognised the role of CAFs in the progression of various tumour types, including head and neck cancer [7,8]. CAFs were found to provide cancer cells with several growth factors and chemokines that encourage tumour growth and invasion, as well as metastasis through remodelling of the extracellular matrix [9,10,11].

Studies have shown that CAFs induce epithelial-to-mesenchymal transition and thus, invasiveness of oral cancer cells [12,13]. Another research study revealed that CAFs participate in recruiting and inducing tumour-associated macrophages in the stroma of oral squamous cell carcinoma, thus increasing the immunosuppressive environment [14]. A CAF-rich TME has been found to be associated with increased mortality in a study conducted on 77 patients with tongue cancer [15]. Among the various growth factors and cytokines that are produced by CAFs, TGF-β (Transforming growth factor beta), EGF (Epidermal growth factor), and chemokines were found to play a major role in inducing proliferation and invasion of cancer cells [16,17].

The treatment of head and neck squamous cell carcinoma (HNSCC) is set to undergo rapid changes, as novel treatment targets informed by genomic profiling and novel molecular targeted therapies continue to make progress. The first molecular targeting therapy to demonstrate a survival advantage for patients with HNSCC has emerged in the context of EGFR (Epidermal growth factor receptor) biology. Cetuximab, a monoclonal antibody against EGFR, is the only approved targeted therapy for head and neck squamous cell carcinoma (HNSCC). Treatment efficacy of Cetuximab, however, is low, with an objective response rate of 13% in the monotherapy setting [18,19] and 36% in combination with chemotherapy [20]. Patients who respond to Cetuximab eventually develop resistance. The PI3K (Phosphoinositide-3-kinase)-Akt (v-Akt murine thymoma viral oncogene) pathway is activated downstream of EGFR, TGFβR, and the chemokine receptor and is emerging as potentially one of the most important pathways in HNSCC. PIK3CA (Phosphatidylinositol-4,5-bisphosphate 3-kinase catalytic subunit alpha) is the most frequently mutated oncogene for HNSCC (approximately 20%) and may play a role in both HPV-negative and HPV-positive tumours. Multiple therapeutic strategies targeting PI3K are being explored, and multiple agents either alone or in combination are in development [21,22,23].

The class 1 PI3ks are a set of lipid kinases that phosphorylate the relatively abundant membrane phospholipid, phosphatidylinositol 4,5 biphosphate (PIP2), generating small quantities of phosphatidylinositol 3,4,5 triphosphate (PIP3). This latter lipid signal controls a diverse set of effector molecules, including the Akt group of oncogenic kinases (also known as protein kinase B) [24]. Activation of Akt, a 60-kDa serine/threonine kinase, depends on PI3K [25]. An increase of cellular PIP3 by PI3K eventually allows the activation of Akt by phosphorylation at residues T308 and S473 [26]. This activation is completed by structural modification stimulated by PI3K-dependent kinase-1 (PDK-1)-dependent phosphorylation at T308 and stabilisation by mTORC2 (Mammalian target of rapamycin complex 2)-dependent phosphorylation at S473 [27]. Full activation of Akt is achieved once it is phosphorylated at the Thr 308 and Ser 473 residues [28]. The serine/threonine kinase, Akt, is responsible for cell proliferation, differentiation, as well as cell motility and migration [29,30].

In this study, we aimed to investigate the role of cancer-associated fibroblasts in the stimulation of head and neck cancer cell migration and invasion by developing a novel 3-D in vitro tumour microenvironment model and identifying the key signalling pathways that might be involved in fibroblast-induced head and neck cancer metastasis.

## 2. Results

### 2.1. Conditioned Medium Collected from Fibroblasts Induced Cancer Cell Scattering in an Akt-Dependent Manner

**Figure 1:**
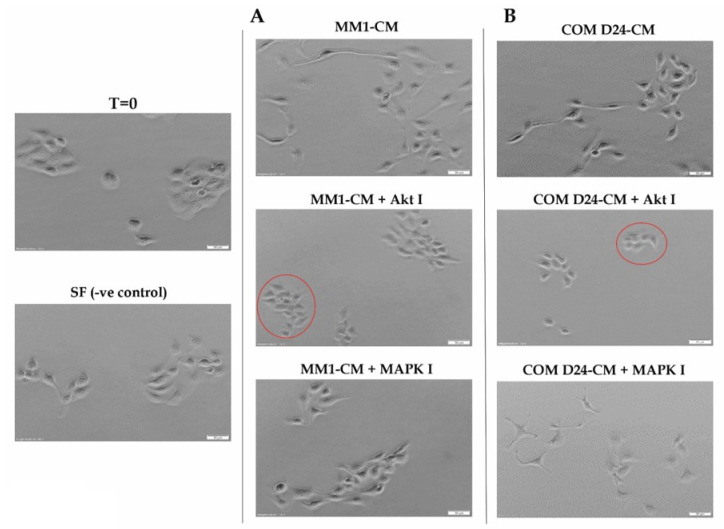
Scattering of TYS cells in response to fibroblast CM (conditioned medium) with or without inhibitors. (**A**) TYS cells treated with MM1 conditioned medium ± inhibitors. Cancer cells scattered out from the colonies in response to MM1 conditioned medium and only the Akt inhibitor blocked the scattering of TYS cells (compact colonies, red circle). (**B**) TYS cells treated with COM D24 conditioned medium ± inhibitors. Cancer cells scattered out from the colonies (mesenchymal-type cells) in response to COM conditioned medium and only the Akt inhibitor blocked the scattering of TYS cells (compact colonies, red circle). Scattering of the cancer cells was observed for 48 h and images of the cells captured using an inverted microscope with a 10× objective lens and processed with CellSense 2.0 imaging software (Olympus). T = 0 represents the baseline cells before adding the test conditions and cells treated with SF-MEM (Serum-free MEM medium) were regarded as a negative control.

Conditioned medium collected from both MM1 and COM D24 cells induced oral adenoid cancer cell scattering after 48 h. Cancer cells scattered out from the compact colonies and their cuboidal shape changed to spindle mesenchymal-type cells in response to conditioned medium from both cell lines. Cells treated with serum-free medium (negative control) did not scatter (Figure 1). MK2206, an Akt inhibitor, completely blocked the scattering of cancer cells induced by conditioned medium from both fibroblast cell lines. However, inhibitors of MAPK (Mitogen activated protein kinase) (Figure 1), EGFR(Epidermal growth factor receptor), TGFβRI (Transforming growth factor receptor 1), and the CXCR4 (C-X-C chemokine receptor) (Figure S3) did not inhibit conditioned medium-induced cancer cell scattering.

### 2.2. Conditioned Medium from Oral Cancer-Associated Fibroblasts (COM D24) Activated Akt More Than the Negative Control in Scattered Cancer Cells

**Figure 2:**
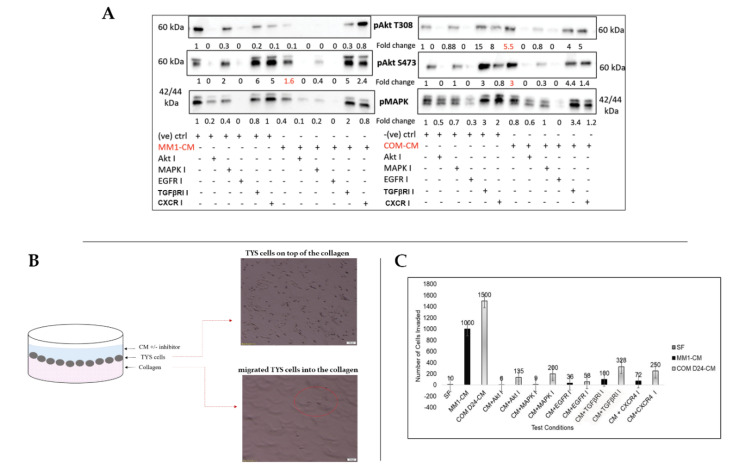
Activation of Akt and MAPK in scattered TYS cells and migration of TYS into the matrix. (**A**) Phosphorylated Akt at Threonine 308 (pAkt T308) and Serine 473 (pAkt S473) and phoshorylated42/44-MAPK in TYS cells after 48 h of the scatter assay. COM-CM activated Akt in scattered TYS cells (red font) more than the negative control and MM1-CM. Fibroblast CM did not activate MAPK in TYS cells after 48 h. Blots were normalised against total protein, quantified, and the data expressed as the fold change compared to the negative control. SF-MEM-treated TYS cells in the scatter assay after 48 h were regarded as the negative control. (**B**) Graphical representation of the collagen gel migration assay in which TYS cells were plated on top of collagen gels followed by treatment with CM ± inhibitors. Cells that had migrated (red circle) into the collagen were counted manually by selecting 5 random areas within the gel after 48 h. (**C**) Bar graph representing the number of TYS cells that migrated into the matrix in response to fibroblast CM with or without inhibitors. COM-CM stimulated TYS cell migration into the matrix more than the negative control (SF-MEM-treated cells) and that of MM1-CM. The Akt and EGFR inhibitors were the most effective inhibitors in blocking CM-induced TYS cell migration.

Conditioned medium collected from cultures of MM1 and COMD24 cells activated Akt as indicated by phosphorylation of S473 in the scattered TYS cells 1.6 and 3 times more than that of serum-free medium from negative control cells, respectively. However, MM1-CM did not activate Akt by phosphorylation of the T308 residue, but COM D24-CM activated Akt at T308 in scattered cancer cells 5.5 times more than that negative control cells. MAPK (Mitogen activated protein kinase), in comparison, was not activated by any of the CM samples (compared to the negative control). The Akt inhibitor completely blocked the activation of Akt in cancer cells treated with conditioned medium. However, inhibitors for TGFβRI and the chemokine receptor (CXCR4) did not block the activation of Akt at either residue in the COMD24-CM-induced scattered TYS cells (Figure 2A). Full blot images are included in the Figures S1 and S2.

### 2.3. COM D24-CM and MM1-CM Stimulated Cancer Cell Invasion into the Collagen Matrix

After observing that conditioned medium (CM) collected from fibroblasts could induce TYS cell scattering/migration, we examined whether the CM could induce TYS cell invasion into a collagen matrix treated with/without the inhibitors (Figure 2B). Both MM1-CM and COM D24-CM stimulated TYS cells to invade into the matrix 100 times and 150 times more than the negative control (SF), respectively. COM D24-CM induced TYS cell invasion 1.5 times more than that of MM1-CM. The Akt inhibitor blocked MM1-CM-induced cancer cell invasion (by 99%) effectively and COM-CM-induced invasion (by 91%) partially as more cells were still invaded than the control. The MAPK inhibitor blocked MM1-CM-induced cancer cell invasion effectively but blocking of COM-CM-induced cancer cell invasion was partial, as 20 times more cells still invaded than the control. However, the EGFR inhibitor blocked MM1-CM- and COM-CM-induced cancer cell invasion effectively. Blocking of MM1-CM- and COM-CM-induced cancer cell invasion by both the TGFβ receptor inhibitor and CXCR4 inhibitor were also not effective (Figure 2C).

### 2.4. Fibroblast Conditioned Medium Stimulated Cancer Cell Evasion from the Spheroids into the Collagen Matrix More Than the Control

**Figure 3:**
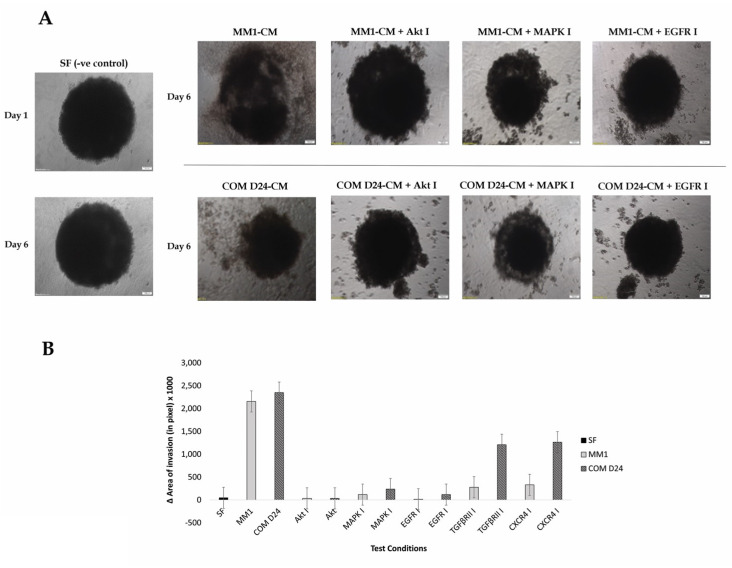
TYS cells invasion from their spheroids in response to fibroblast CM ± the inhibitors. (**A**) TYS spheroids were embedded into a collagen matrix followed by treatment with fibroblast CM with or without the inhibitors. Images of the invasive TYS cells from the spheroids were captured after day 6 using an inverted microscope with a 4X objective lens. Images of the TYS spheroids treated with TGFβRI inhibitor and CXCR4 inhibitor are presented in the Supplementary Material (Figure S4). SF-MEM-treated TYS spheroids were regarded as a negative control. Scale bar =100 µm. (**B**) Graphical representation of TYS invasion into the collagen matrix from the spheroids in response to fibroblast CM ± the inhibitors after day 6. COM-CM stimulated TYS invasion from the spheroids into the matrix, more than the negative control (SF-MEM-treated cells) and MM1-CM. The Akt and EGFR inhibitors were the most effective inhibitors in blocking CM-induced TYS invasion. The area of invasion (out from the edge of the spheroid) was calculated using ImageJ software.

Cell invasion from the spheroids into the surrounding matrix appeared as spindle-like projections, whereas there were no such projections observed where invasion had not been stimulated. There was no change in the area of invasion of TYS spheroids observed in serum-free medium and this was used as a negative control (Figure 3A). TYS cells into the collagen matrix from their spheroids invaded after day 6, in response to both MM1 and COM D24 conditioned medium. The inhibitors for Akt and MAPK completely blocked cancer cell invasion from the spheroids induced by both MM1-CM and COM-CM (Figure 3). The EGFR inhibitor also completely blocked CM-induced invasion of cancer cells from the spheroids into the matrix (Figure 3). However, inhibitors of TGFβRI and CXCR4 did not block COM-CM-induced invasion of cancer cells from the spheroids (Figure 3B and Figure S4).

### 2.5. COM D24 Cells Stimulated Cancer Cells to Invade from the Spheroids into the Collagen Matrix and the Akt Inhibitor Completely Blocked This Invasion

**Figure 4:**
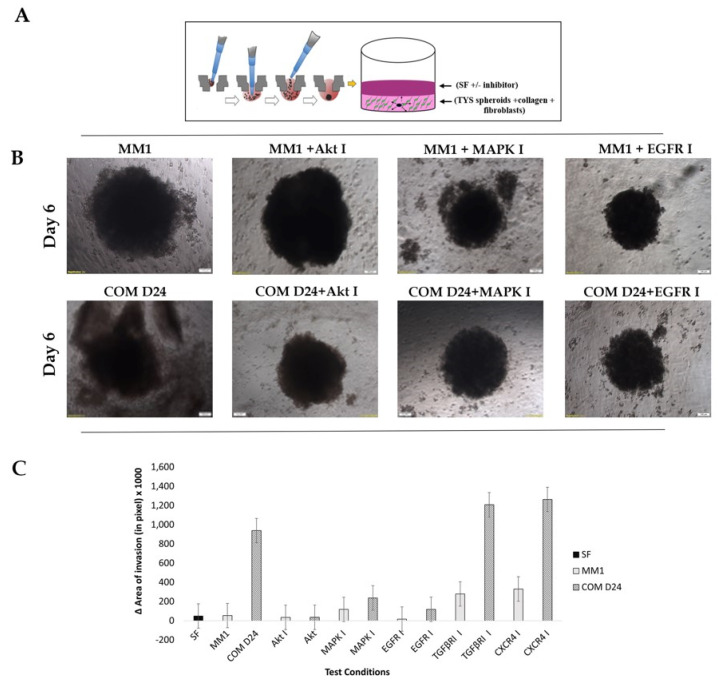
TYS cells’ invasion from the spheroids in response to fibroblasts ± inhibitors. (**A**) A 3-D TME model that illustrates the development of 3-D TYS spheroids by the hanging drop method, adding fibroblasts and embedding the spheroids into collagen. (**B**) TYS invasion from the spheroids in response to fibroblasts with or without exogenous inhibitors. Images of the invasive TYS cells from the spheroids were captured after day 6 using an inverted microscope with a 4X objective lens. Images of the TYS spheroids treated with TGFβRI and CXCR4 inhibitors are presented in the Supplementary Material (Figure S5). SF-MEM-treated TYS spheroids without the fibroblasts were regarded as the negative control (the same as Figure 3A). TYS cells invaded into the matrix from the spheroids in response to COMD24 cells. Scale bare = 100 µm (**C**) Graphical representation of TYS cell invasion into the matrix from the spheroids in response to fibroblasts ± inhibitors after day 6. COM D24 cells stimulated TYS cells’ invasion from their spheroids more than the negative control (without fibroblasts) and that of MM1 cells. The Akt and EGFR inhibitors were the most effective inhibitors in blocking COM D24 cell-induced TYS cell invasion. However, TGFβRI and CXCR4 inhibitors did not block TYS invasion. The area of invasion (out from the edge of the spheroid) was calculated using ImageJ software.

Figure 4A illustrates a simplistic 3D model of tumour microenvironment (TME) comprising of TYS spheroids, collagen and fibroblast cells. COM D24 cells stimulated TYS cell invasion from the spheroids into the collagen (Figure 4B) 19-fold more than the negative control (SF-MEM-treated spheroids) (Figure 4C). Inhibition of the TGFβ receptor and chemokine receptor by their respective inhibitors resulted in stimulation of the invasion of TYS cells from the spheroids by about 23-fold compared to that of the negative control (Figure S5 and Figure 4C). The Akt inhibitor completely blocked COM D24-induced invasion of TYS cells from the spheroids into the matrix (Figure 4B,C). The MAPK inhibitor and EGFR inhibitor also blocked COM D24-induced cancer cell invasion from the spheroids but not as efficiently as the Akt inhibitor (Figure 4B,C). MM1 cells did not stimulate TYS cell invasion from the spheroids into the collagen, in comparison to the negative control. COM D24 cells stimulated invasion 17-fold more in comparison to MM1 cells (Figure 4B,C).

### 2.6. EGFR Inhibitor Was Toxic to Akt-Silenced Oral Adenoid Cancer Cells

**Figure 5:**
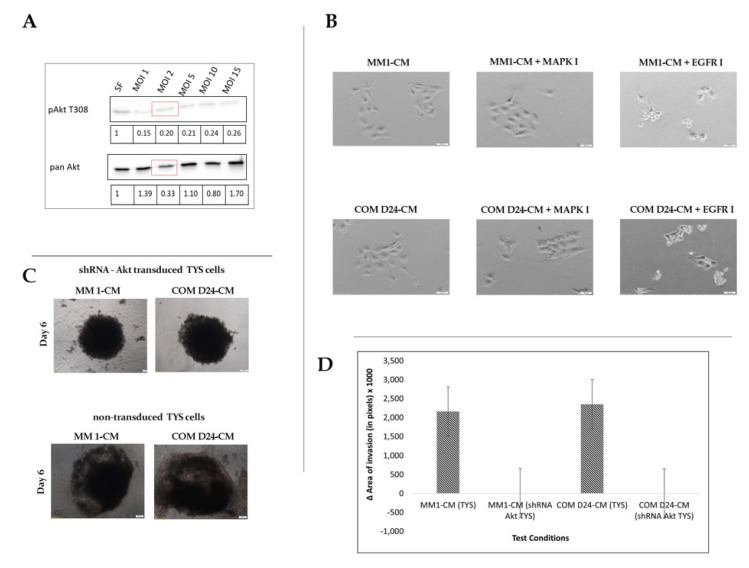
Scattering and invasion of Akt-silenced TYS cells. (**A**) Akt expression and activation after silencing the Akt gene using differentmultiplicity of infection (MOI) of shRNA lentivirus. MOI 2 (red box) was chosen for transfection. Akt-silenced TYS cells were called shRNA Akt TYS. (**B**) Scattering of shRNA Akt TYS cells in response to fibroblast conditioned medium. shRNA Akt TYS cells were not stimulated to scatter in response to fibroblast CM and did not survive when treated with EGFR inhibitor after 48 h. Scale bar = 50 µm (**C**) Invasion of shRNA Akt TYS cells from the spheroids into the collagen matrix in response to fibroblast CM. Images were captured using an inverted microscope (Olympus) and processed with CellSense 2.0 imaging software (Olympus). Scale bar = 100 µm (**D**) Graphical presentation of Akt-silenced Akt TYS invasion and shRNA Akt TYS cells were not stimulated to invade in response to fibroblast CM.

TYS cells were transfected with shRNA (Short hairpin ribonucleic acid) Akt at different multiplicity of infection (MOI). The cell lysates from the cells transfected at MOI 1, 2, 5, 10, and 15 were blotted against pAkt and Pan Akt antibodies and compared with the negative control. MOI 2 was chosen for transfection as the phosphorylation of Akt T308, and total Akt was observed to be considerably less than that of the negative control. Akt-silenced TYS cells were then regarded as shRNA Akt TYS cells (Figure 5A). Akt-silenced TYS cells were not stimulated to scatter in response to conditioned medium, whereas non-transfected TYS cells were. Cell death was noted when Akt-silenced TYS cells were treated with the EGFR inhibitor (Figure 5B). After 6 days of the 3-D spheroid assay, it was observed that the Akt-silenced TYS cells did not invade into the collagen from their spheroids when treated with either MM1-CM or COM D24-CM, as compared to the non-transfected TYS cells (Figure 5C). Akt-silenced TYS cells lost the ability to invade into the collagen matrix completely, in comparison to non-transfected cells treated with MM1-CM and COM-CM (Figure 5D).

## 3. Discussion

CAFs are considered to play a crucial role in tumour progression. A recent study revealed that CAFs trigger tumour progression in OSCC by secreting various growth factors to the nearby cells [31], as observed in this study. The normal fibroblasts (NFs) that are found in the tumour microenvironment can interact with cancer cells and become ‘activated’, leading to CAF formation. Thus, both NF and CAF interaction with cancer cells may promote tumour progression by activating migration and invasion [32]. Both the NF and the CAFs stimulated migratory behaviour of the oral adenoid cancer cells (TYS) in the 2-D scatter assay. These assay results did not show any clear difference in the migratory behaviour of the cancer cells in response to NF (MM1) and CAF (COM-D24), thus further invasion experiments were conducted. Invasion assays concluded that CAF-CM stimulated oral adenoid cancer cell invasion more than NF-CM. The 3-D spheroid invasion assay confirmed upregulation of CAF-CM- or CAF-mediated cancer cell invasion, in comparison to that of normal fibroblasts. Thus, to conclude, CAFs have a pivotal role in migration and invasion in head and neck cancer. During tumour progression, despite producing and secreting various growth factors, CAFs play a major role in the dysregulated collagen turnover that leads to tumour fibrosis. Tumour fibrosis is characterised by excessive collagen deposition in the tumour surroundings. Collagens directly affect the hallmarks of cancer by regulating cell proliferation, differentiation, gene expression, migration, invasion, and metastasis [33,34,35]. Most of the collagens, especially fibroblast-derived collagens (types I, II, III, V, VI, XI), are upregulated in cancer at both the gene and protein levels, thus modulating critical steps in tumourigenesis [33]. Collagen type I, III, and XI expression is highly increased in HNSCC, with almost no expression in healthy controls [36,37] and knockdown of type XI collagen significantly decreases proliferation, invasion, and migration compared to controls [33,37]. Collagen has also been shown to affect metastasis by increasing invasiveness in pancreatic and ovarian cancers [38,39]. Thus, the 3-D TME model containing fibroblasts and collagen, developed in this research, can be a useful tool to study the relationship between the tumour microenvironment and metastasis.

Akt phosphorylation, both at Thr 308 and Ser 473, was upregulated in CAF-CM-treated cancer cells, whereas only Ser 473 was upregulated in NF-CM-treated cancer cells, compared to the negative control. This may explain why the CAF-CM-treated cancer cell invasion was higher than that of the normal fibroblasts. This also proves the long-standing theory that Akt needs to be phosphorylated at both residues to be fully active, stable, and induce certain bioactivities. Through multivariate studies it has been reported that Akt activation is a significant independent prognostic indicator for OSCC [40]. It is evident from this study that the Akt inhibitor is responsible for blocking both CAF- and NF- induced cancer cell migration and invasion. An in vitro study conducted by Knowles et al. in 2011 showed that MK-2206 (Akt inhibitor) efficiently blocked HNSCC chemotaxis and migration [41]. Phase I clinical trials of MK-2206 in combination with Erlotinib (EGFR inhibitor) showed early evidence of antitumour activity in advanced solid tumours [42]. Activation of MAPK transforms normal cells to tumour cells, causing a high risk of developing a second primary tumour in oral cancer, and distant metastasis is also observed in advanced stages [43]. A higher rate of MAPK phosphorylation was observed in CAF-treated cancer cells, in comparison to the normal fibroblasts, supporting the theory that MAPK might also be responsible for CAF-mediated metastasis in oral cancer. Though the MAPK inhibitor and EGFR inhibitor blocked the invasion of cancer cells in response to NF and CAF, these inhibitors did not block scattering in 2-D cell cultures. The Akt inhibitor, however, blocked the migration and invasion of cancer cells effectively in both the 2-D and 3-D assays. This data indicates that Akt is a vital molecule to study in order to ascertain the underlying signalling mechanisms involved in fibroblast-induced head and neck cancer metastasis. TGFβ has a dual nature as it exhibits both pro- and antitumour effects and helps in the acquisition of the cancer associated fibroblast (CAF) phenotype. Many TGFβ antibodies, kinase inhibitors, and antisense oligonucleotides are being assessed for efficacy in phase III trials [8]. TGFβ has been shown to activate Akt, leading to cell migration in prostate cancer [44]. TGFβ1 stimulates the phosphorylation of SMAD (Sma and Mad proteins), MAPK, and Akt in head and neck cancer but stimulated the migration of cancer cells in an Akt-dependent manner [45]. In prostate cancer, CXCR4 is overexpressed, and a loss of the tumour suppressor PTEN was reported to activate Akt and regulate the CXCL12/CXCR4 signalling pathway in metastasis [46]. CXCR4 expression was also found to be upregulated in human oesophageal squamous cell carcinoma and modulated cell migration and invasion by Rho-A (Ras homolog family member A), Rac-1 (Ras related C3 botulinum toxin substrate 1), and Cdc42 (Cell division control protein 42 homolog) through Akt-dependent mechanisms [47]. CAFs are also reported to promote invasion through the CXCR4 pathway in gastric cancer and a CXCR4 antagonist blocked the invasiveness of gastric cancer [48]. Thus, the literature suggests that TGFβ and the chemokine receptor might have a role in cancer metastasis. The data presented here indicate that inhibitors of TGFβR and CXCR4 do not block CAF- and NF-induced cancer cell migration and invasion, since they did not block Akt phosphorylation but rather increased it. This unexpected result and the mechanism of action of these two inhibitors needs to be explored further. Furthermore, silencing the Akt gene in oral adenoid cancer cells blocked scattering and invasion, even when treated with CAF and NF conditioned medium. It was also observed that blocking both Akt and EGFR leads to cell death, which can be studied further to check its specificity to cancer cells.

## 4. Materials and Methods

### 4.1. Antibodies and Inhibitors

Details of the antibodies and inhibitors used in this study are included in the Table 1. 

### 4.2. Cell Culture

This studydid not require any human/animal subjects to acquire any ethical approval. The adenoid squamous carcinoma cell line (TYS), derived from a minor salivary gland, was a kind gift from Dr. Koji Harada, University of Tokushima, Japan. Normal oral mucosal fibroblasts (MM1) and oral cancer-associated fibroblasts (COM D24) were isolated in-house from explant cultures of biopsies from the Oral Surgery Clinic, Ninewells Hospital, Dundee Cells were cultured and maintained in minimum essential medium (MEM) supplemented with 10% (*v*/*v*) foetal calf serum and 200 mM glutamine and incubated at 37 °C in a humidified incubator with 5% CO_2_. Prior to growth of the MM1 and COM D24 cell lines, culture dishes were coated with collagen 1 (#C-3867, Sigma, St. Louis, MO, USA).

### 4.3. Conditioned Medium Preparation

MM1 and COM D24 cells were cultured in 9-mm dishes until 70–80% confluent. The cells were then washed three times with 4 mL of phosphate buffered solution (PBS), followed by a final wash with 4 mL of serum free-MEM medium (SF-MEM). The cells were then maintained in 5 mL of SF-MEM and incubated for two days. The medium was then collected, centrifuged for 5 min at 900 RPM, filtered using a 0.2-µm filter, and stored at −20 °C.

### 4.4. Cell Scatter Assay

The cell scatter assay was used to investigate the scattering (motility) of TYS cells out from compact colonies, in response to MM1 and COM D24 conditioned medium. Here, 40% COM D24-CM and 25% MM1-CM were found to be the best dilutions, after optimisation. TYS cells were plated in 60-mm dishes, at a density of 1 × 10^4^ cells/mL. Once the cells had formed small colonies (10–15 cells/colony) after 24 or 48 h, CM with or without inhibitors was added and the colonies observed for 48 h. Assays were repeated at least three times.

### 4.5. Cell Lysis, SDS-PAGE, and Western Blot

Scattered TYS cells were washed twice with ice-cold PBS, then ice-cold RIPA (Radioimmunoprecipitation assay) buffer containing both phosphatase inhibitors (# 04906837001, Roche, Bavaria, Germany). and protease inhibitors (#A32963, Thermo Scientific, Rockford, IL, USA) Cells were incubated on ice for 10 min and the cell lysates were then collected.

For SDS-PAGE and Western blot, lysates were mixed with an equal volume of Laemmli loading buffer (Bio-Rad, Hercules, CA, USA) containing 5% (*v*/*v*) 2-Mercaptoethanol and then heated at 95 °C for 5 min, followed by centrifugation. Then, 20 μL of each sample were loaded into 10-well 10% SDS PAGE BioRad pre-cast gels. After SDS-PAGE, protein bands were transferred from the gel onto PVDF (Polyvinylidene difluoride) membrane, blocked with blocking buffer (1% *w*/*v* dried milk in 1x Tris buffered solution with Tween-20), and then incubated with primary antibody overnight at room temperature, followed by incubation with secondary antibody. Finally, blots were developed with BioRad Clarity Western ECL Substrate, and chemiluminescence was detected using a GelDoc system (BioRad, Hercules, CA, USA). Bands on the blots were then normalised against the total protein and quantified using Image lab software (BioRad, Hercules, CA, USA).

### 4.6. 3-D-Collagen Gel Assay

Collagen gels (2 mg/mL) were made by mixing collagen 1 (#C-4243, Sigma, St. Louis, MO, USA) with 10XMEM medium and 7.5% (*w*/*v*) sodium bicarbonate and incubated for 1 h to allow complete polymerisation. Then, TYS cells were plated on the top of the gels at a density of 2 × 10^4^ cells/well in a 48-well plate, and the plate was then incubated for 4 h to allow cell attachment. After this, the medium was discarded and conditioned medium with or without the inhibitors was added in the wells and incubated for 48 h. Serum-free MEM was added to some wells and regarded as the negative control. Five areas were chosen randomly inside the gel in each well and pictures were taken of the migrated cells. The mean number of migrated cells per well was calculated and the results were compared to the negative control. The experiments were carried out three times.

### 4.7. 3-D Spheroid Invasion Assay

A 96-well hanging drop plate (#HDP 1096, Perfecta3D^®^ hanging drop plate, 3D Biomatrix Inc., Ann Arbor, MI, USA) was used to form spheroids from TYS cells. TYS cells grown in 2-D dishes were trypsinised, collected by centrifugation, and then re-suspended in 10% (*v*/*v*) FCS-MEM. Cells were then plated at a density of 1 × 10^6^ cells/mL in the hanging drop plate (as instructed by the manufacturer’s protocol), and the medium was changed every day. After 72 h, cell aggregation and spheroid formation were observed and confirmed under the microscope.

Then, the spheroids were transferred into the collagen solution prepared as described in Section 4.6 (before polymerisation) by pipetting the hanging droplets with 50 µL of serum-free medium, followed by incubation for 1 h. Test conditions (conditioned medium ± inhibitors) were then added onto the top of the gel and incubated for 6 days. The test conditions were replaced every 2 days by adding 50 µL of each condition to the well. SF-MEM medium was also added and regarded as a negative control.

MM1 or COM D24 cells was added to the collagen matrix solution at a concentration of 2.5 × 10^5^ cells/mL. Then, the spheroids were transferred into the collagen/fibroblast mix and incubated for 1 h for polymerisation. Test conditions (SF-MEM medium ± inhibitors) were then added onto the top of the gel and incubated for 6 days. The test conditions were replaced every 2 days by adding 50 µL of each condition to the well.

Images of the spheroids and invasive cells were taken using an inverted microscope (IX70, Olympus, Tokyo, Japan) and processed by CellSense software (Olympus). The maximum invasive areas were measured using ImageJ software (NIH) and compared to the spheroid area (the values quoted are in pixel measurements).

### 4.8. Gene Silencing Using Akt 1 shRNA (Human) Lentivirus

shRNA lentivirus transfection was carried out according to the manufacturer’s instructions (Santa Cruz Biotechnology, Dallas TX, USA). All the reagents related to the transfection were purchased from Santa Cruz Biotechnology. TYS cells were plated in a 6-well plate at a concentration 1 × 10^5^ per well and incubated overnight. On the next day, a mixture of 10% (*v*/*v*) FCS-MEM growth medium with polybrene (#sc-134220) at a final concentration 5 μg/mL was prepared. The growth medium on the cells was then replaced with 1 mL of polybrene-medium mixture per well. The TYS cells were then transfected by adding the required volume of shRNA Akt (#sc-29195-v) or shRNA control lentiviral particles (#sc-108080) to the medium after determining the multiplicity of infection (MOI). On the third day, the polybrene-culture medium mix was replaced with 1 mL of 10% (*v*/*v*) FCS-MEM growth medium and incubated overnight. On the fourth day, the cells were split into 60-mm dishes at a 1:3 ratio and incubated for 24 h. On the fifth day, the stable clones were selected by the addition of 300 ng/mL puromycin dihydrochloride (#sc-108071). Fresh puromycin-containing medium was added every 3–4 days, until antibiotic-resistant colonies were identified. Silencing of Akt expression was confirmed by Western blot assay using both pAkt and Pan Akt antibodies. Transfected cells were denoted as shRNA Akt TYS and used in the scatter and 3-D spheroid invasion assay to investigate the effect of the fibroblast conditioned medium and inhibitors on the migration and invasion of Akt-silenced cancer cells.

## 5. Conclusions

The 3-D model of the tumour microenvironment developed in this project could be used as a valuable tool to study the association between TME and metastasis. Data from this project suggests that cancer -associated fibroblasts stimulate oral adenoid cancer cell migration and invasion in a PI3K/Akt signalling pathway-dependent manner. This study also suggests that an Akt inhibitor alone or in combination with the EGFR inhibitor should be investigated further to prove their efficacy as a targeted therapy in metastatic HNSCC.

## Figures and Tables

**Table 1:** Details of the antibodies and inhibitors used in this study.

Name	Catalogue/Ref No.	Company and Address	Dilution/ Conc. Used
Primary antibodies			
Phospho-Akt (Thr308) (C31E5E) Rabbit mAb	2965	Cell Signaling Tech., Denver, MI, USA	1:1000
Phospho-Akt (Ser473) (D9E) XP Rabbit mAb	4060	Cell Signaling Tech., Denver, MI, USA	1:2000
Akt (pan) (C67E7) Rabbit mAb	4691	Cell Signaling Tech., Denver, MI, USA	1:1000
Phospho- MAPK 42/44 Rabbit mAb	9101	Cell Signaling Tech., Denver, MI, USA	1:2000
Secondary antibodies			
Goat anti-rabbit IgG, HRP-linked	7074	Cell Signaling Tech., Denver, MI, USA	1:2000
Chemical inhibitors			
MK2206 (Akt inhibitor)	S1078	Selleckchem, Houston, TX, USA	5 µM
Gefitinib (EGFR inhibitor)	4765S	Cell Signaling Tech., Denver, MI, USA	10 µM
PD98059 (MAPK inhibitor)	9900L	Cell Signaling Tech., Denver, MI, USA	50 µM
TGFβRI kinase inhibitor VII	616458	Calbiochem, San Diego, CA, USA	5 µM
AMD3100 Octahydrochloride hydrate (CXCR4 Inhibitor)	A5602	Cell Signaling Tech., Denver, MI, USA	1 µM

## Supplementary Materials

The following are available online at https://www.mdpi.com/2072-6694/12/8/2093/s1, Figure S1: Full blot images of the pAkt T308, pAkt S473 and pMAPK treated with MM1-CM, Figure S2: Full blot images of the pAkt T308, pAkt S473 and pMAPK treated with COM-CM, Figure S3: TYS cells scattering in response to fibroblast CM with EGFR, TGFβRI and CXCR4 inhibitors, Figure S4: TYS cells invasion from their spheroids in response to fibroblast CM with the TGFβRI and CXCR4 inhibitors, Figure S5: TYS cell invasion from the spheroids in response to fibroblasts, with the TGFβRI and CXCR4 inhibitors.

## Abbreviation

CAF	Cancer Associated Fibroblasts
TME	Tumour Microenvironment
CM	Conditioned Medium
MAPK	Mitogen Activated Protein Kinase
EGFR	Epidermal Growth Factor Receptor
TGFβR	Transforming Growth Factor beta Receptor
CXCR4	C-X-C Chemokine Receptor 4
HNSCC	Head and Neck Squamous Cell Carcinoma
