# Supplementary material for: Head and Neck Cancer Metastasis and the Effect of the Local Soluble Factors, from the Microenvironment, on Signalling Pathways: Is It All about the Akt?
Source: Cancers (Basel). 2020 Jul 28;12(8):2093. doi: 10.3390/cancers12082093 (PMC7463947; doi:10.3390/cancers12082093)
Supplement: Supplementary file 1 [file cancers-12-02093-s001.pdf]

Supplementary Materials

Head and neck cancer metastasis and the effect of the local soluble factors, from the microenvironment, on signalling pathways. Is it all about the Akt?

Hanan Ahmed, Arpa Ghoshal, Sarah Jones, Ian Ellis and Mohammad Islam

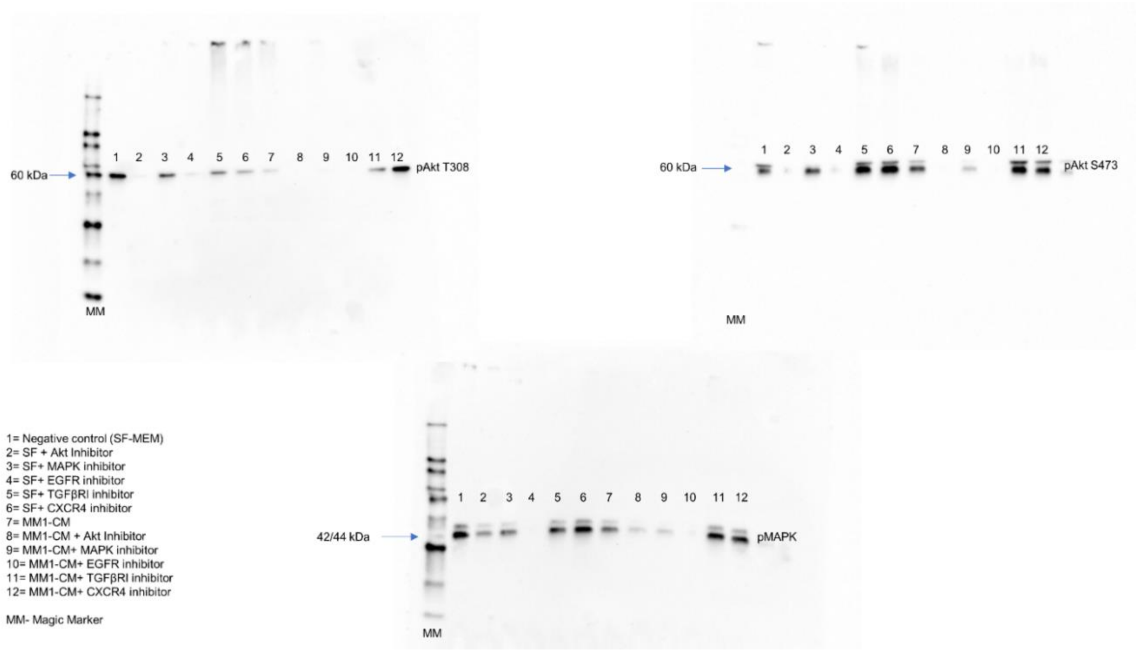

Figure S1. Full blot images of the pAkt T308, pAkt S473 and pMAPK treated with MM1-CM. TYS cells were treated with MM1 conditioned medium with or without the inhibitors for 48 h. SF-MEM treated cells were regarded as negative control.

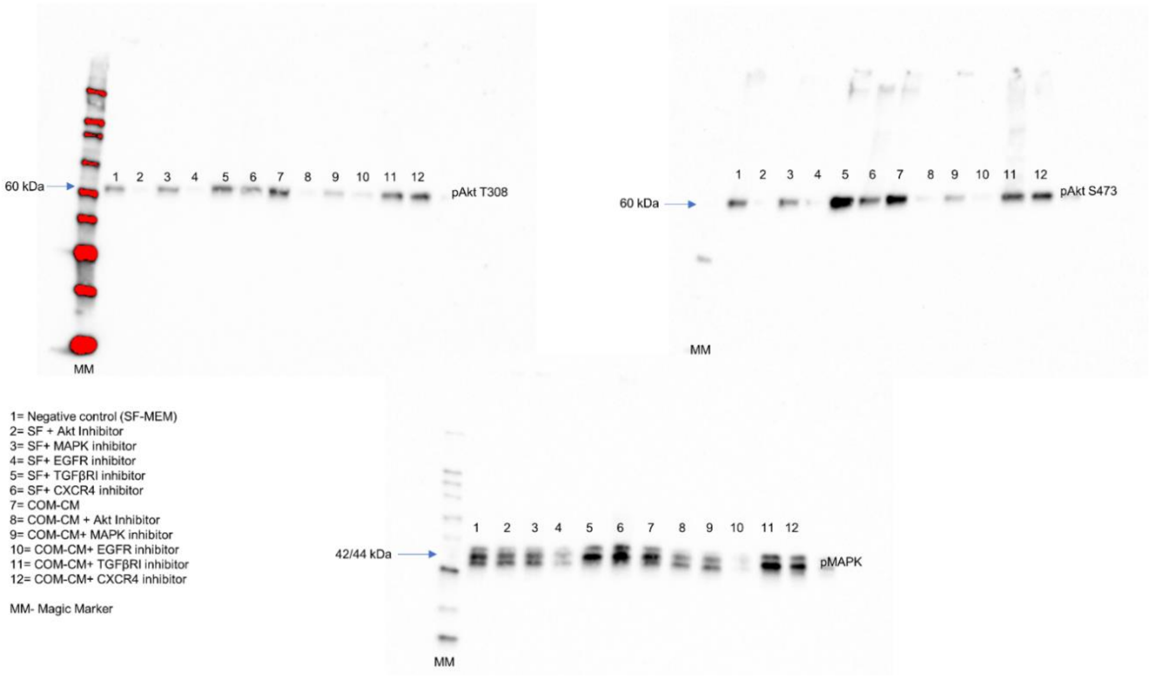

Figure S2. Full blot images of the pAkt T308, pAkt S473 and pMAPK treated with COM-CM. TYS cells were treated with COM conditioned medium with or without the inhibitors for 48 h. SF-MEM treated cells were regarded as negative control.

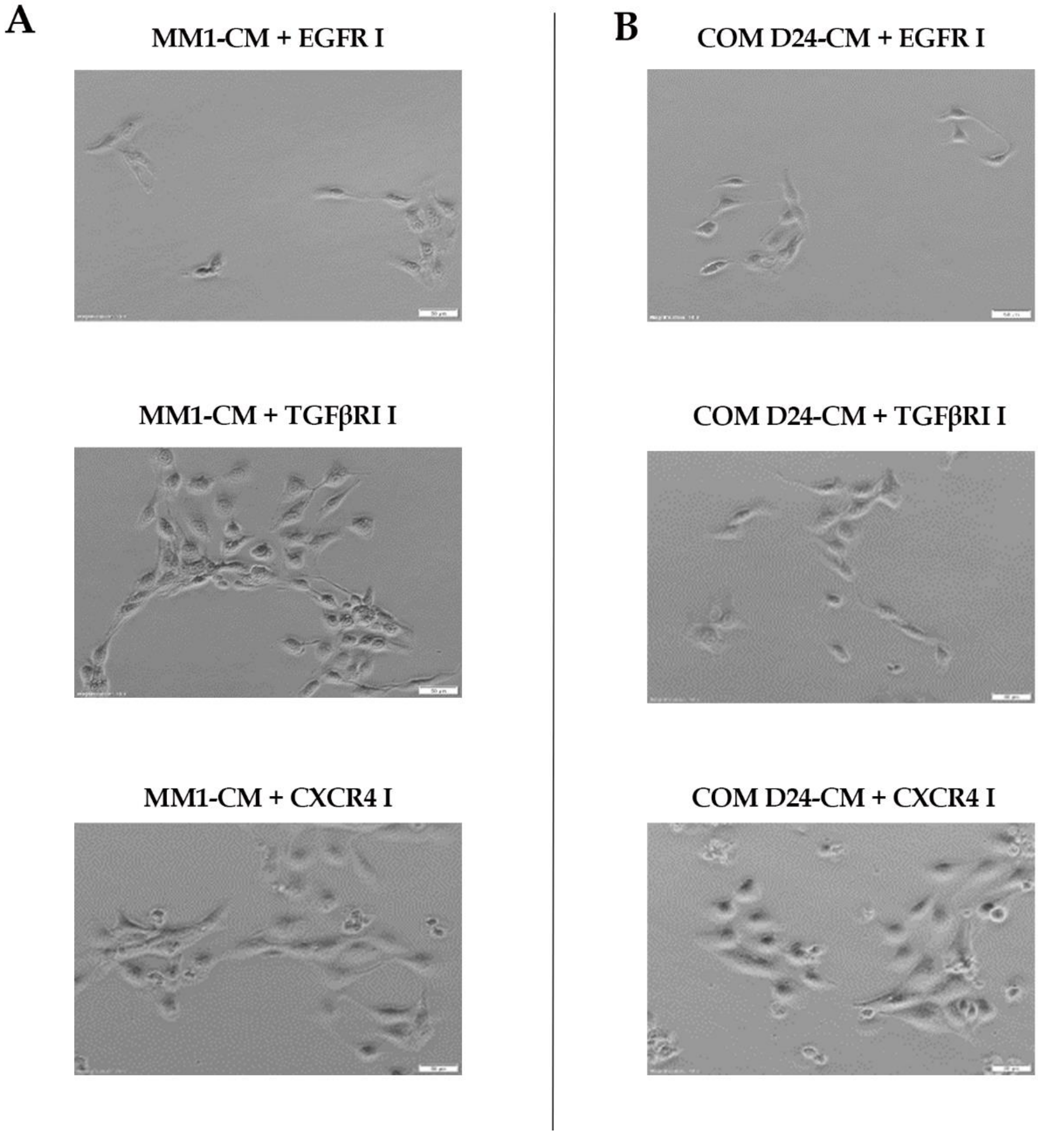

Figure S3. TYS cells scattering in response to fibroblast CM with EGFR, TGFβRI and CXCR4 inhibitors. (**A**) TYS cells treated with MM1-CM for 48 hours in combination with the inhibitors of EGFR, TGFβRI and CXCR4. (**B**) TYS cells treated with COM D24-CM for 48 hours in combination with the inhibitors of EGFR, TGFβRI and CXCR4. None of the inhibitors blocked the fibroblast CM-induced scattering of TYS cells. Scale bar = 50 μm.

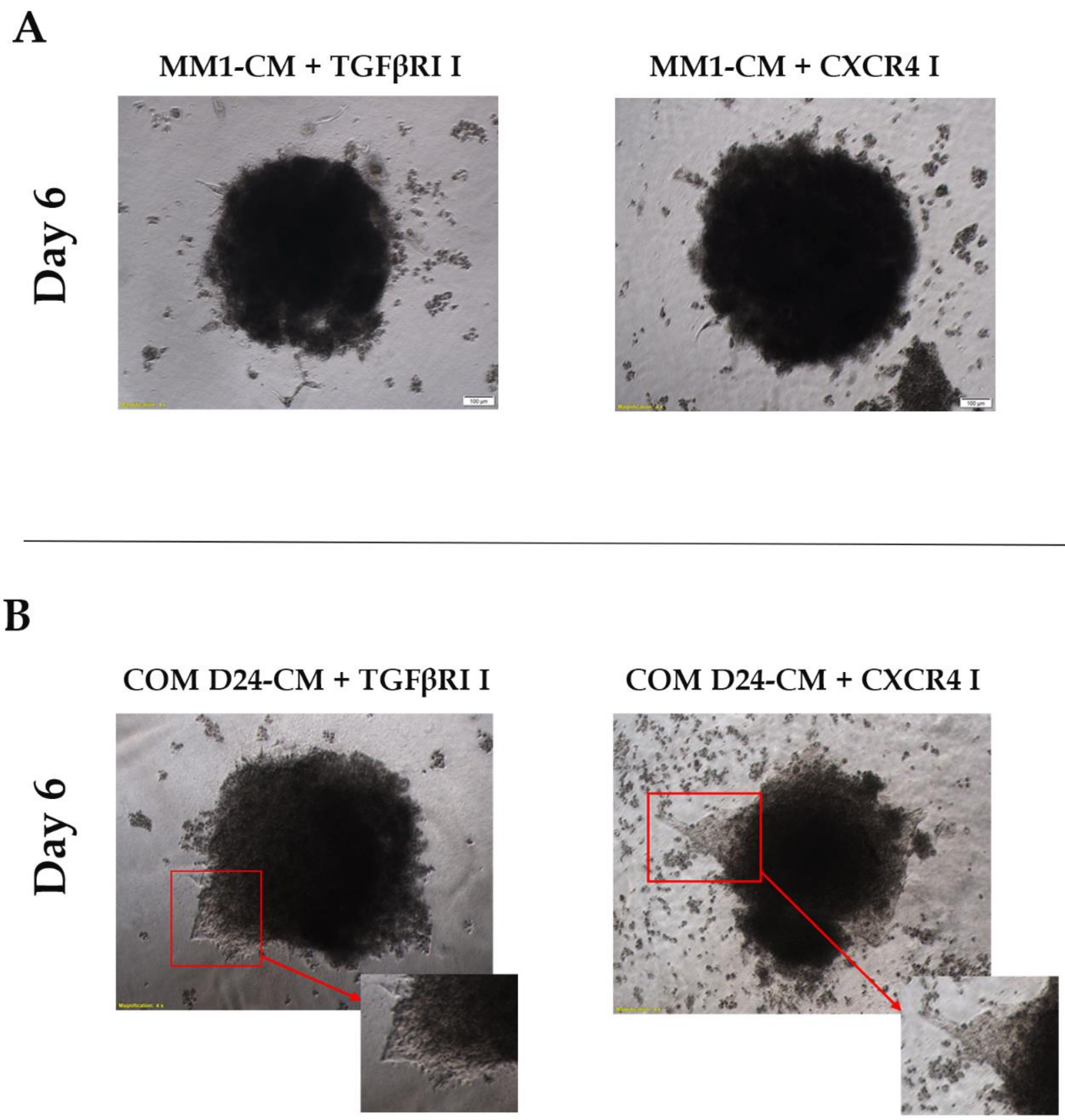

Figure S4. TYS cells invasion from their spheroids in response to fibroblast CM with the TGFβRI and CXCR4 inhibitors. (**A**) MM1-CM treatment of TYS spheroids in combination with the TGFβRI and CXCR4 inhibitors for 6 days. (**B**) COM D24-CM treatment of TYS spheroids in combination with the TGFβRI and CXCR4 inhibitors for 6 days .TGFβRI and CXCR4 inhibitors blocked MM1-CM induced TYS cell invasion but did not block COM D24-CM induced TYS cells invasion. Scale bar = 100 μm.

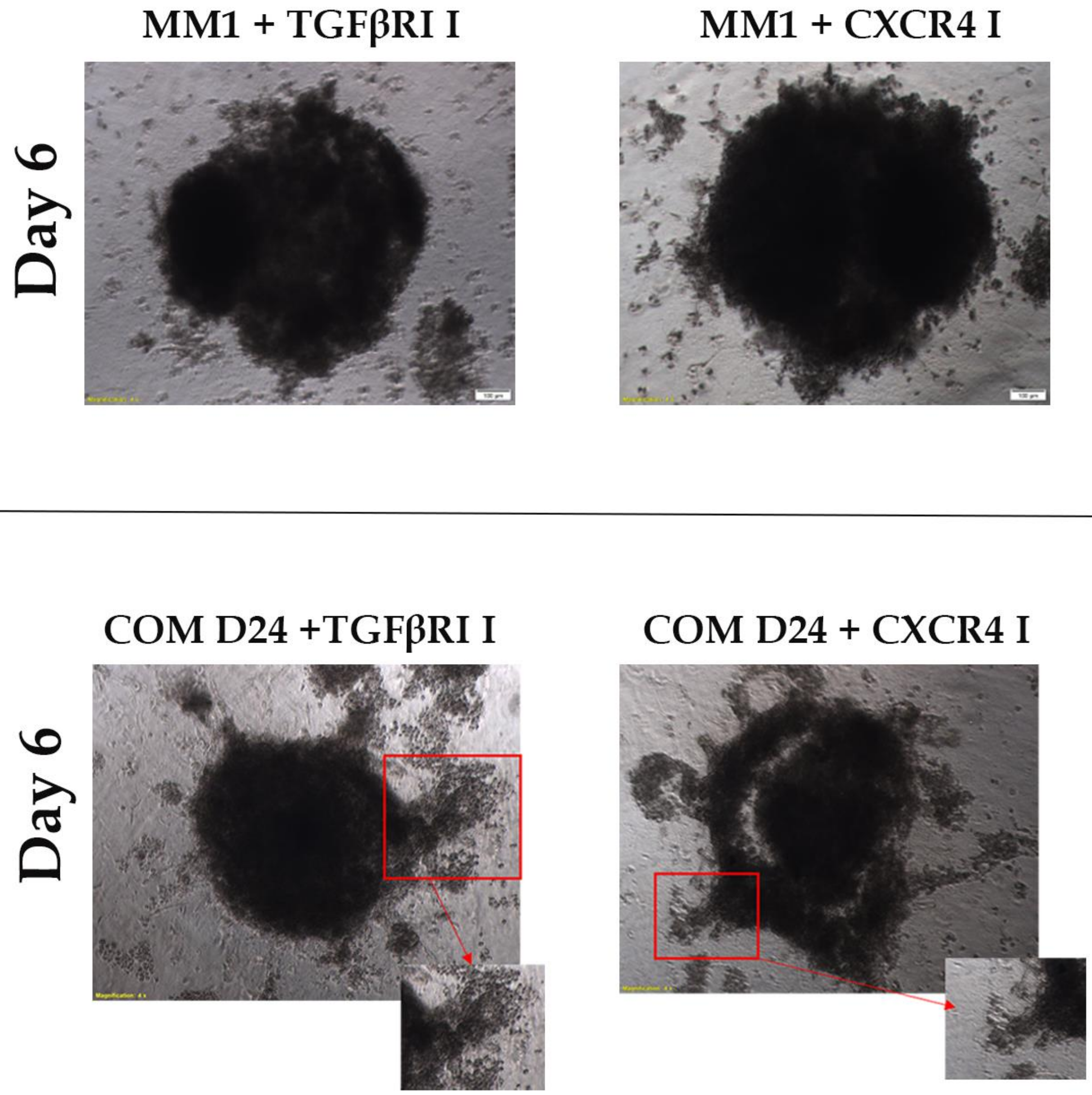

Figure S5. TYS cell invasion from the spheroids in response to fibroblasts, with the TGFβRI and CXCR4 inhibitors. TGFβRI and CXCR4 inhibitors did not block COM D24-induced TYS cell invasion, but blocked MM1 cells induced TYS cells invasion.

© 2020 by the authors. Licensee MDPI, Basel, Switzerland. This article is an open access article distributed under the terms and conditions of the Creative Commons Attribution (CC BY) license (http://creativecommons.org/licenses/by/4.0/).
